# Environmental variables associated with *Nothophaeocryptopus gaeumannii* population structure and Swiss needle cast severity in Western Oregon and Washington

**DOI:** 10.1002/ece3.5639

**Published:** 2019-09-12

**Authors:** Patrick I. Bennett, Jeffrey K. Stone

**Affiliations:** ^1^ Department of Botany and Plant Pathology Oregon State University Corvallis OR USA

**Keywords:** environmental adaptation, evolutionary divergence, forest pathology, population genetics, Swiss needle cast

## Abstract

The environment has a strong influence on the abundance and distribution of plant pathogenic organisms and plays a major role in plant disease. Climatological factors may also alter the dynamics of the interactions between plant pathogens and their hosts. *Nothophaeocryptopus* (=*Phaeocryptopus*) *gaeumannii*, the causal agent of Swiss needle cast (SNC) of Douglas‐fir, is endemic to western North America where it exists as two sympatric, reproductively isolated lineages. The abundance of this fungus and the severity of SNC are strongly influenced by climate. We used statistical and population genetic analyses to examine relationships between environment, pathogen population structure, and SNC severity. Although *N. gaeumannii* Lineage 2 in western Oregon and Washington was most abundant where SNC symptoms were most severe, we did not detect a significant relationship between Lineage 2 and disease severity. Warmer winter temperatures were inversely correlated with foliage retention (*AFR*) and positively correlated with the relative abundance of Lineage 2 (*PL2*). However when distance inland, which was strongly correlated with both *AFR* and *PL2*, was included in the model, there was no significant relationship between Lineage 2 and *AFR*. Spring/early summer dew point temperatures also were positively associated with total *N. gaeumannii* abundance (colonization index (*CI*)) and inversely correlated with *AFR*. Warmer summer mean temperatures were associated with lower *CI* and higher *AFR*. Our results suggest that the two lineages have overlapping environmental optima, but slightly different tolerance ranges. Lineage 2 was absent from more inland sites where winters are colder and summers are warm and dry, while Lineage 1 occurred at most sites across an environmental gradient suggesting broader environmental tolerance. These relationships suggest that climate influences the abundance and distribution of this ecologically important plant pathogen and may have played a role in the evolutionary divergence of these two cryptic fungal lineages.

## Graphical abstract

This study was designed to test hypotheses about the relationships between the environment, *Nothophaeocryptopus gaeumannii* population structure, and the severity of an important Douglas‐fir disease, Swiss needle cast. This ecological system was investigated by combining data from genetic markers with environmental data and disease severity data. The results of this study suggest that two reproductively isolated lineages of this fungal pathogen have differing environmental optima, but does not support previous studies suggesting that the lineages differ in their aggressiveness.

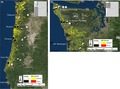

## 1 INTRODUCTION

Climate plays a major role in influencing the geographic distributions of plant pathogens and their hosts and may lead to changes in host–pathogen dynamics (Sturrock et al., 2011). Climate change is predicted to directly affect pathogens most strongly, and fungi causing foliage diseases in particular are most likely to be influenced by environmental variables (Harvell et al., 2002). Outbreaks of damaging forest pathogens such as *Dothistroma septosporum*, which causes foliage loss, growth reductions, and mortality of native and introduced *Pinus* species, have been linked to regional weather patterns (Kliejunas et al., 2009; Welsh, Lewis, & Woods, 2014). The recent emergence of this major forest disease and others around the globe may be attributable, at least in part, to climate change (Harvell et al., 2002; Kliejunas et al., 2009; Welsh et al., 2014; Woods, Coates, & Hamann, 2005; Woods et al., 2016). Microbial pathogens and insects, because of their shorter generation times, respond more rapidly to environmental changes than forest communities and may amplify the effects of climate change on forest health (Kliejunas et al., 2009). Changes to forest disturbance regimes due to the influences of climate on the interactions between disease, fire, and insects are expected to impact the future ecological trajectories of forested landscapes (Agne et al., 2018; Sturrock et al., 2011).

Swiss needle cast (SNC) is a foliar disease specific to Douglas‐fir (*Pseudotsuga menziesii* (Mirb.) Franco) caused by the ascomycete *Nothophaeocryptopus* (=*Phaeocryptopus*)* gaeumannii* (T. Rohde) Videira, C. Nakash., U. Braun & Crous. The inhibition of gas exchange and CO_2_ assimilation resulting from the cumulative occlusion of stomata by the pseudothecia of *N. gaeumannii* causes premature needle abscission (Manter, Bond, Kavanagh, Rosso, & Filip, 2000; Manter, Winton, Filip, & Stone, 2003). Impaired carbon assimilation and reduced photosynthetic capacity due to foliage loss result in height and diameter growth reductions of 20%–50% relative to healthy trees (Maguire, Kanaskie, Voelker, Johnson, & Johnson, 2002; Maguire, Mainwaring, & Kanaskie, 2011; Manter et al., 2003). The physiological stress caused by this disease rarely leads to mortality, as even the most severely diseased trees retain the current year's foliage (Hansen et al., 2000; Maguire et al., 2002). However, the loss of vigor associated with SNC may diminish the ability of Douglas‐fir to compete with other conifer species (e.g., *Tsuga heterophylla*), thereby altering the ecology and successional trajectory of Douglas‐fir in mixed natural stands (Lee et al., 2017; Stone, Coop, & Manter, 2008; Zhao, Maguire, Mainwaring, & Kanaskie, 2014).

Although the disease was first described from a Douglas‐fir plantation in Switzerland in 1925, *Nothophaeocryptopus gaeumannii* is presumed to be native to the Pacific Northwest (Hansen et al., 2000) where the population is subdivided into two reproductively isolated lineages (Bennett & Stone, 2016; Winton, Hansen, & Stone, 2006). Lineage 1 occurs throughout the natural range of Douglas‐fir and worldwide where Douglas‐fir is grown as an exotic (Winton et al., 2006). Lineage 2 has a more restricted distribution (Bennett & Stone, 2016; Winton et al., 2006) and is most abundant along the western Coast Ranges in Oregon and Washington within a few kilometers of the coast. The abundance of Lineage 2 decreases further inland, where it is often supplanted entirely by Lineage 1 (Bennett & Stone, 2016; Winton et al., 2006). The factors influencing the spatial distributions of these lineages are not currently understood. Previous studies have identified strong genetic differentiation between these two lineages (Bennett & Stone, 2016; Winton et al., 2006). Despite the fact that there is circumstantial evidence for reproductive isolation between the two lineages, there is currently insufficient evidence to describe them as distinct biological or phylogenetic species.

Prior to the late 1970s, SNC had not caused any serious damage in Pacific Northwest forests (Hansen et al., 2000). However, symptoms of SNC have been intensifying in this region since 1990s, and a 2016 aerial survey identified approximately 200,000 hectares of affected land in Oregon (Hansen et al., 2000; Ritóková et al., 2016). Its proliferation and subsequent emergence as a threat to Douglas‐fir forest health and productivity in western North America is thought to have been perpetuated by the widespread planting of Douglas‐fir where the climate is particularly conducive to disease in a narrow band of low‐elevation coastal forests along the western slopes of the Coast Ranges in Oregon and Washington that has historically been considered the *Picea sitchensis* vegetation zone (Franklin & Dyrness, 1973; Hansen et al., 2000).

Because the distribution of Lineage 2 in the western Coast Ranges in Oregon and Washington corresponds to the region where the most severe SNC symptoms (foliage loss and growth reduction) have been documented, a causal relationship between Lineage 2 and the recent intensification and expansion of SNC in this region has been suggested (Winton et al., 2006). Compared to Lineage 1, recovery of Lineage 2 isolates from diseased foliage was twice as likely in severely diseased stands, and only half as likely in healthier stands (Winton et al., 2006). In coastal Oregon, Douglas‐fir stands with higher relative proportions of Lineage 2 had more sparse canopies, suggesting less foliage retention due to SNC (Winton et al., 2006). Visual estimates of foliage discoloration as an indicator of disease severity also suggested a causal relationship with the relative abundance of Lineage 2 in the stand (Winton et al., 2006). Isolates of *N. gaeumannii* collected from sites with severe disease also seemed to cause more severe SNC symptoms in an inoculation study, suggesting that more virulent or aggressive genotypes may be more prevalent in the low‐elevation coastal forests in Oregon and Washington where SNC is most severe (Winton, 2001). These observations informed our hypotheses about the potential relationships between the distribution of *N. gaeumannii* Lineage 2 and SNC severity.

The regional climate in the western Coast Ranges in Oregon and Washington has a strong influence on the distribution and severity of SNC, and variation in *N. gaeumannii* abundance and SNC symptom severity in relation to site‐specific climate factors has been well documented (Coop & Stone, 2010; Hansen et al., 2000; Lee, Beedlow, Waschmann, Burdick, & Shaw, 2013; Lee et al., 2017; Manter, Reeser, & Stone, 2005; Rosso & Hansen, 2003; Stone, Coop, et al., 2008; Stone, Hood, Watt, & Kerrigan, 2007; Watt, Stone, Hood, & Palmer, 2010; Zhao, Maguire, Mainwaring, & Kanaskie, 2012; Zhao, Mainwaring, Maguire, & Kanaskie, 2011). Winter temperature consistently has been identified as being strongly correlated with *N. gaeumannii* abundance, and thus SNC severity, given that needle colonization and the development of pseudothecia continue throughout winter following the spring/summer infection period (Capitano, 1999; Manter et al., 2005; Stone, Capitano, & Kerrigan, 2008). Colder winter temperatures limit the colonization of needles by *N. gaeumannii* (Manter et al., 2005). Leaf wetness and free moisture (as precipitation, fog, or dew) during the spring and early summer are necessary for spore dispersal, adhesion, and germination on the needle surface (Capitano, 1999; Manter et al., 2005), and thus are highly influential in predictive spatial models of SNC severity when severity estimates are based on the abundance of *N. gaeumannii*. Temperatures above 30°C inhibit the growth of *N. gaeumannii*, and thus, warmer average summer temperatures also are associated with less severe SNC symptoms (Capitano, 1999; Lee et al., 2017; Rosso & Hansen, 2003; Zhao et al., 2011). Predictive models based on combinations of these factors explain much of the variability (*R* ^2^ = 57%–78%) in SNC severity in western Oregon and Washington forests (Lee et al., 2017; Manter et al., 2005; Rosso & Hansen, 2003; Stone, Coop, et al., 2008). A trend of increasing mean winter temperatures and spring precipitation in the Pacific Northwest in recent decades has resulted in conditions conducive to the intensification and expansion of SNC (Abatzoglou, Rupp, & Mote, 2014; Lee et al., 2017; Stone, Coop, et al., 2008).

Our objectives for this study were to (a) assess the spatial distributions of the two *N. gaeumannii* lineages in relation to SNC severity in the Oregon (OR) and Washington (WA) Coast Ranges, (b) determine whether any relationship exists between SNC severity and the relative abundance of *N. gaeumannii* Lineage 2, and (c) examine the relationships between the genetic structure of *N. gaeumannii* populations, SNC severity, and key environmental variables with a multivariate statistical ordination. Given previous observations of the relationship between the spatial distribution of *N. gaeumannii* Lineage 2 and SNC severity, we aimed to test the hypothesis that the relative abundance of Lineage 2 within sites is positively correlated with disease severity. The analyses performed here were also designed to test the hypothesis that SNC severity and the spatial distributions of the two lineages (and thus the spatial genetic structure of *N. gaeumannii* populations) are correlated with environmental variables that have been previously identified as being critical for the development of *N. gaeumannii*.

## 2 MATERIALS AND METHODS

### 2.1 Foliage sampling

**Figure 1:**
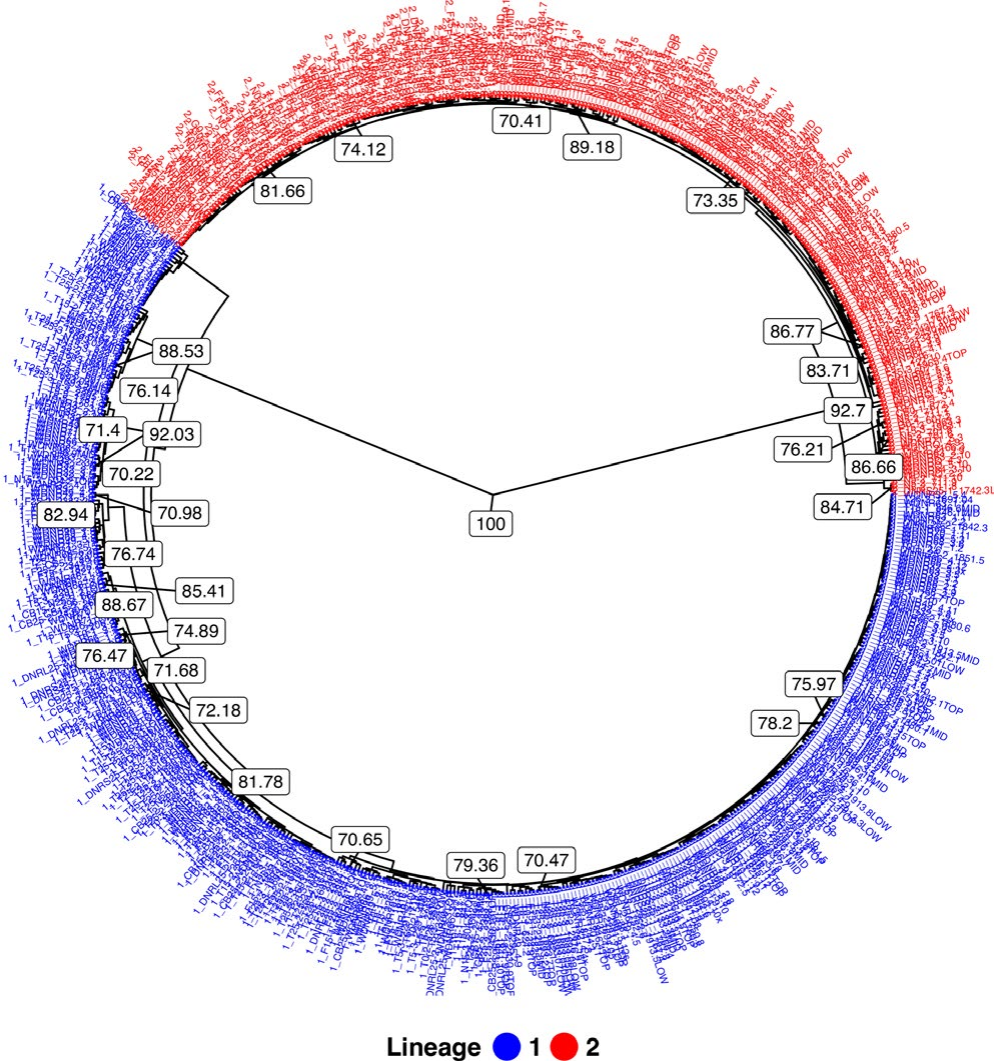
Genetic distance dendrogram (UPGMA, unweighted pair‐group method with arithmetic means) from bootstrap analysis of Nei's genetic distance showing divergence between genotypes corresponding to two *Nothophaeocryptopus gaeumannii* lineages from Oregon and Washington (clone‐censored, *N* = 663). Node labels represent bootstrap statistics (≥ 70%) from 10,000 replicate trees

Douglas‐fir foliage was collected from 23 sites in the western Oregon Coast Range in a plot network maintained by the Oregon State University Swiss Needle Cast Cooperative (SNCC) (Ritóková et al., 2016; Shaw, Filip, Kanaskie, Maguire, & Littke, 2011). For these sites, the sampling was concurrent with annual disease assessments performed 2014–2016. In 2015, foliage was also collected from 11 sites in Washington managed by the Washington Department of Natural Resources (WA DNR) (Figure 1). With the exception of the WA DNR sites in southwest Washington and those near Gold Beach, OR, the sampling sites were generally arranged in transects stratified by distance inland, with sites ranging from the shoreline to 56 km inland (Figure 1). At each site, foliage was collected from second‐ and third‐year internodes on secondary branches in the upper crowns of five randomly selected 10‐ to 30‐year‐old Douglas‐fir trees. From one of the five trees sampled at each of the SNCC sites, foliage samples were also collected from the lower, middle, and upper crowns to assess within‐tree diversity. The foliage was stored on ice and promptly returned to the campus of Oregon State University for storage in a cold room for no longer than 7 days prior to processing.

### 2.2 Isolation of *N. gaeumannii* from Douglas‐fir Foliage

The protocol for isolating *N. gaeumannii* from infected Douglas‐fir needles is described in detail in Bennett and Stone (2016). Briefly, needles with pseudothecia of *N. gaeumannii* were attached to the lids of Petri dishes with double‐sided adhesive tape, placed over water agar, and incubated for 48–72 hr. Individual ascospores were removed from the agar with sterilized forceps and transferred onto 2% malt agar (MA) (Difco Laboratories). Cultures were incubated at 18°C for a minimum of 2 months, and often up to 6 months.

### 2.3 Molecular techniques

The protocols for DNA extraction, PCR amplification, and genotyping of simple sequence repeats (SSRs) are described in Bennett and Stone (2016). The DNeasy Plant Kit (Qiagen) was used to extract genomic DNA from vegetative mycelium. The manufacturer's protocol was modified with the addition of a maceration procedure (Bennett & Stone, 2016). For each isolate, ten SSR loci (Winton, Stone, & Hansen, 2007) were amplified in three multiplexed PCR reactions (Bennett & Stone, 2016). The Qiagen Type‐It Microsatellite PCR kit (Qiagen, Hilden, Germany) and its associated protocols were used, but with reaction volumes of 12.5 µl. The amplification was performed with the thermal cycling protocol described in Bennett and Stone (2016).

The PCR amplicons were submitted to the Center for Genome Research and Biocomputing (CGRB, Oregon State University) for SSR genotyping via capillary electrophoresis with an ABI 3730 DNA Analyzer (Applied Biosystems, Thermo Fisher Scientific Corporation). Allele sizes and genotypes were assigned with ABI GeneMapper 4.1 (Applied Biosystems, Thermo Fisher Scientific Corporation) and were also visually examined for each locus and isolate to confirm accuracy. A positive control isolate was included with each PCR and genotyping run to ensure that the results were consistent and reproducible. One of the loci, Pgdi5, did not amplify reliably for all isolates and therefore was omitted from the dataset prior to analysis. The actual number of trees per site represented by the isolates in the SSR dataset was often less than five due to a number of steps that may have resulted in sample loss, such as variation in the presence of pseudothecia, contamination during isolation and culturing, and inconsistent amplification during PCR. The genotypes used for this study included those analyzed in Bennett and Stone (2016) (i.e., those sampled in 2014) along with additional isolates that were collected in 2015 and 2016.

### 2.4 Data analysis

All statistical and population genetic analyses were performed with the R statistical computing software version 3.4.1 (R Core Team, 2017). The multilocus genotypes (MLGs) consisting of alleles for the 9 SSR loci were formatted in Microsoft Excel 2016 with GenAlEx 6.503 (Peakall & Smouse, 2012, 2006). The MLGs were then imported into R version 3.4.1 (R Core Team, 2017) with the R package *poppr* version 2.5.0. The MLGs were organized in a stratified population hierarchy that included levels for lineages, states within lineages, sites within states, and trees within sites.

Estimates of genetic diversity, including genotypic diversity (Shannon‐Weiner index, *H*) and Nei's unbiased gene diversity (expected heterozygosity, *H*_e_) (Nei, 1978), were calculated with the R package *poppr* 2.5.0 (Kamvar et al., 2015, 2014). The Shannon‐Weiner diversity index, *H*, was estimated from 1,000 iterations of a bootstrap analysis with rarefaction. Genotypic richness was estimated as the number of expected multilocus genotypes (eMLG) with a rarefaction sample size ≥ 10 (Kamvar et al., 2014).

The membership of each isolate in either Lineage 1 or Lineage 2 was visualized as a UPGMA dendrogram constructed from 10,000 bootstrap replicates of Nei's genetic distance (Nei, 1978). This analysis was performed with the R packages *poppr* 2.5.0 (Kamvar et al., 2015, 2014) and visualized with *ggtree* version 1.12.0 (Yu, Smith, Zhu, Guan, & Lam, 2017). Genetic differentiation between the two *N. gaeumannii* lineages was estimated by calculating a standardized differentiation metric, *G*′_ST_ (Hedrick, 2005), with the R package mmod (Winter, 2012). This measure is scaled such that the value for two populations that do not share any alleles is one, and the value for population pairs that share all alleles is equal to zero (Hedrick, 2005). This metric is also corrected for the observed population heterozygosity and the number of subpopulations being compared (Hedrick, 2005; Winter, 2012). If we assume that the rate of gene flow between populations is higher than the mutation rate within populations, indices such as *G*′_ST_ can provide a reasonable estimate of migration (Balloux & Lugon‐Moulin, 2002; Hedrick, 2005).

Aerial disease survey data were obtained from the Oregon Department of Forestry (https://www.oregon.gov/ODF/ForestBenefits/Pages/ForestHealth.aspx) (Ritóková et al., 2016), and from the Washington Department of Natural Resources (Ramsey, Omdal, Dozic, Kohler, & Boderck, 2015). Researchers with the Oregon State University Swiss Needle Cast Cooperative (SNCC, http://sncc.forestry.oregonstate.edu/) and the Washington Department of Natural Resources provided estimates of both average foliage retention (*AFR*) and colonization index (*CI*) for sites in OR and WA (Ramsey et al., 2015; Ritóková et al., 2016). These disease severity metrics were measured using methods similar to those described in Manter et al. (2005) and Watt et al. (2010). For this study, *AFR* was expressed as the average percentage of foliage remaining across four needle age classes from secondary branches collected in the midcanopies of 10 trees from each site. An estimate of the average percentage of stomata occluded by pseudothecia, *CI*, was calculated as the product of incidence (the proportion of needles bearing pseudothecia, *N* = 50 needles) and the percentage of stomata occluded by pseudothecia (*N* = 10 needles). The percentage of stomata occluded was calculated by averaging the numbers of pseudothecia from 100 stomata in each of three sections per needle (base, middle, and tip) for each sample of ten 2‐year‐old needles from each of three canopy sections per tree (lower, middle, and upper) for ten trees from each site.

Pearson correlation coefficients were calculated for all pairwise correlations between geographic and disease variables (for the 34 sites for which disease severity data were available) with the R package *Hmisc* version 4.1‐1 (Harrell, 2017). Scatter plots associated with these statistical analyses were constructed with *ggplot2* (Wickham, 2016). To account for the possibility that Douglas‐fir foliage retention, SNC severity, and the relative proportion of *N. gaeumannii* Lineage 2 may vary independently along the west–east sampling gradient (due to climatic and other spatial geographic factors), a linear mixed model was utilized to investigate the influence of the proportion of Lineage 2 on *AFR* with the distance inland from the coast held at its mean value (26 km). Distance inland is related to continentality and serves as a proxy for a complex combination of environmental variables (i.e., average temperature, precipitation, RH, dew point deficit, fog, wind speed, and other unknown factors) that are expected to influence Swiss needle cast severity and foliage retention (Zhao et al., 2011). The following model was designed to test the null hypothesis that the proportion of Lineage 2 had no effect on *AFR* after accounting for distance inland (*H*_0_: *β*_1_ = 0):

$$Y_{i}=\beta_{0}+\beta_{1}X_{1i}+\beta_{2}X_{2i}+\varepsilon_{i}$$

where *Y_i_* is the average foliage retention of the *i* ^th^ site. The intercept *β*_0_ is the mean average foliage retention when the relative proportion of Lineage 2 recovered at the site is 0 and the distance inland is 0 km, *β*_1_ is the coefficient for the effect of the relative proportion of Lineage 2 on mean average foliage retention, *X*_1_ *_i_* is the relative proportion of Lineage 2 recovered from the *i* ^th^ site, *β*_2_ is the coefficient for the effect of the distance inland (km) on mean average foliage retention, *X*_2_ *_i_* is the distance inland (km) of the *i* ^th^ site, and *ε_i_* is the random effect of the *i* ^th^ site on mean average foliage retention, *ε_i_* ~ *N*(0, *σ* ^2^).

Nonmetric multidimensional scaling (NMDS) was used to visualize genetic differentiation between each of the sample sites. Roger's euclidean genetic distance (Rogers, 1972) was calculated pairwise between each of the sampling sites with the R package *adegenet* (Jombart, 2008). The NMDS ordination based on this genetic distance matrix was performed with the function *metaMDS* in the R package *vegan* version 2.4‐5 (Oksanen et al., 2017). This method ranks sample units according to their dissimilarity and then attempts to minimize the stress in the relationship between ordination distances and genetic distances (McCune, Grace, & Urban, 2002). Correlations between the ordination, SNC severity, and the environmental/geographic variables associated with each site were calculated with the function *envfit* from the R package *vegan version 2.4-5* (Oksanen et al., 2017). The environmental and geographic overlays were displayed as a series of radiating vectors, with the direction of the vector corresponding to its relationship to the ordination axes, and the length of the vector proportional to the strength of the correlation between the variable and the ordination. Included as vectors on the joint plot were SNC severity (*AFR* and *CI*), the relative proportion of Lineage 2, latitude, longitude, elevation, and several environmental variables related to SNC severity and *N. gaeumannii* abundance (Lee et al., 2017; Manter et al., 2005; Rosso & Hansen, 2003; Stone, Coop, et al., 2008; Stone et al., 2007; Watt et al., 2010; Zhao et al., 2011). Interpolated spatial climatic data were obtained for each of the sample sites from the Parameter‐elevation Regressions on Independent Slopes Model (PRISM) data explorer (PRISM Climate Group, Oregon State University, http://prism.oregonstate.edu).

## 3 RESULTS

### 3.1 Genetic diversity and population structure

This study included a total of 663 unique multilocus genotypes (MLGs) from 1,061 isolates collected from 35 sites in western Oregon and Washington. This includes 492 isolates collected in 2014 (Bennett & Stone, 2016), as well as an additional 579 isolates collected in 2015 and 2016. There was an average of 23.67 alleles per locus, with an average of 0.93% missing data per locus. Total gene diversity (*H*_e_) for the 1,061 isolates was 0.82 (Tables 1 and 2). There were 403 distinct Lineage 1 MLGs and 260 distinct Lineage 2 MLGs (Table 1). The number of private alleles was 88 for Lineage 1 and 55 for Lineage 2. Overall, Lineage 1 had greater genotypic diversity, genotypic richness, and gene diversity than Lineage 2, even after correcting for the difference in sample sizes by rarefaction (Table 1). The two *N. geaumannii* lineages were strongly differentiated (*G*′_ST_ = 0.941) (Table 1). In the UPGMA dendrogram constructed from a bootstrap analysis of Nei's distance, isolates from the two lineages clustered into two distinct groups (Figure 1). The branch lengths and topologies reflected strong genetic differentiation between the lineages and high genetic diversity within each lineage. Of the 657 isolates collected in Oregon, 384 had distinct MLGs, and of the 404 isolates collected in Washington, 282 had distinct MLGs (Table 2). There were 39 private alleles in the Oregon population and 24 in the Washington population.

**Table 1:** Sample sizes and diversity estimates for the two *Nothophaeocryptopus gaeumannii* lineages

	*N*_sites_	*N*_isolates_	MLG^a^	eMLG^b^	*SE* c	*H* d, e	*H*_e_ f	*G*′_ST_ g
Lineage 1	28	646	403	290	5.66	5.48	0.70	
Lineage 2	31	415	260	260	0.00	5.35	0.63	
Total	35	1,061	663	323	6.74	5.66	0.82	0.941

a MLG = number of multilocus genotypes.

b eMLG = expected number of multilocus genotypes in rarefied sample (*n* ≥ 10).

c *SE* = standard error of eMLG estimate.

d *H* = Shannon‐Weiner diversity index.

e Estimated genotypic diversity from 1,000 bootstrap replicates with rarefaction (*n* ≥ 10).

f *H*_e_ = Nei's unbiased gene diversity.

g Hedrick's *G*′_ST_, a standardized measure of population differentiation calculated here to reflect genetic differentiation between Lineage 1 and Lineage 2.

**Table 2:** Site variables, sample sizes, genetic diversity, and disease severity estimates for each of the sites from which Douglas‐fir foliage was collected for isolation of *Nothophaeocryptopus gaeumannii*

Site	Year	Distance inland (km)	Elevation (m)	*N*_trees_	*N*_isolates_	MLG	eMLG	*SE*	*H* *	*H*_e_	L1^a^	L2^b^	CI^c^	AFR (%)^d^
Oregon														
Tillamook														
T0‐1	2014	5.3	134	9	53	36	9.26	0.78	2.2	0.79	26	27	23.1	42.3
T0‐2	2014	4.2	169	5	16	8	5.96	0.91	1.61	0.55	3	13	19.6	52.3
T0‐3	2014	8.3	127	5	26	19	8.79	0.9	2.13	0.75	13	13	24.9	30.5
T5‐3	2014	16.7	158	5	28	24	9.44	0.67	2.23	0.75	22	6	42.8	32.8
T5‐5	2014	17.9	293	5	24	18	8.92	0.83	2.15	0.73	19	5	36.3	46.3
T15‐1	2014	28.1	459	4	49	37	9.27	0.79	2.19	0.75	27	22	16.1	67.1
T15‐2	2014	36.2	576	5	31	25	9.35	0.71	2.21	0.79	17	14	6.7	88.5
T25‐2	2014	49.5	591	5	101	40	8.56	1.03	2.09	0.6	101	0	3.7	88.8
T25‐3	2014	49.2	521	5	63	38	8.93	0.93	2.14	0.67	62	1	6.4	88.5
Newport														
N0‐1	2015	4.7	146	4	28	9	5.85	1.01	1.59	0.56	0	28	22.9	37.3
N5‐2	2015	14.2	457	3	23	17	8.71	0.89	2.12	0.59	0	23	8.8	64
N15‐3	2015	32.3	411	3	13	6	5.3	0.66	1.54	0.66	6	7	8.3	83.2
N25‐5	2015	50.8	251	2	10	6	6	0	1.61	0.65	10	0	9.6	89.3
Florence														
F0‐2	2015	2.7	31	5	11	11	10	0	2.3	0.56	0	11	19.2	43.3
F5‐1	2015	11.2	160	3	13	4	3.73	0.45	1.16	0.49	0	13	12.7	37.8
F15‐1	2014	40.6	595	5	32	21	8.57	0.97	2.09	0.75	28	4	15.3	77.8
F15‐3	2014	38	223	2	15	11	8.12	0.83	2.03	0.68	4	11	17.4	85
F25‐2	2014	50	498	5	38	22	8.07	1.11	2	0.74	25	13	18.5	78.8
Coos Bay														
CB0‐1	2015	8	51	2	11	5	4.73	0.45	1.34	0.32	0	11	29.3	56.5
CB5‐2	2015	25	114	2	8	3	3	0	0.97	0.42	8	0	34.4	57
CB15‐2	2015	42	283	5	12	9	7.82	0.65	1.97	0.82	7	5	17.4	71.3
CB25‐2	2015	57	549	1	23	12	7.32	1.01	1.9	0.75	16	7	4.1	76.5
Gold beach														
G0‐1	2015	1.8	159	3	19	8	6	0.9	1.62	0.58	0	19	18.3	56.5
G0‐2	2016	2.2	98	2	10	6	6	0	1.7	0.52	0	10	21.7	40.5
Washington														
N Olympic Peninsula														
WDNR70	2015	5.9	16	5	37	29	9.05	0.91	2.16	0.78	20	17	21.8	78.9
WDNR71	2015	12.6	105	5	39	28	9.04	0.87	2.17	0.81	21	18	27.2	71.1
WDNR49	2015	20.4	163	5	41	32	9.38	0.72	2.21	0.78	30	11	27	76.7
WDNR68	2015	34.6	116	5	31	27	9.61	0.56	2.25	0.65	29	2	23.5	70.1
WDNR66	2015	43.7	47	5	53	36	8.93	0.94	2.15	0.6	52	1	32.8	60
S Olympic Peninsula														
WDNRQ	2015	4.3	37	5	31	24	9.18	0.79	2.19	0.65	2	29	NA	NA
WDNR64	2015	11.8	67	5	38	26	8.99	0.86	2.16	0.64	2	36	22.4	51.7
WDNR63	2015	21.9	265	5	40	28	9.13	0.82	2.18	0.76	17	23	8.4	75.2
WDNR32	2015	30.6	482	5	46	33	9.1	0.87	2.17	0.64	46	0	14.2	86.5
SW Washington														
DNRL25‐2	2014	58	228	5	25	24	9.85	0.36	2.28	0.74	19	6	18.1	80.3
DNRS25‐1	2014	48	355	5	23	15	8.07	1.01	2.02	0.73	14	9	19.4	78.8
Total				150	1,061	663^e^					646	415		

Abbreviation: NA, data not available.

a L1 = number of *Nothophaeocryptopus gaeumannii* Lineage 1 isolates recovered from the site.

b L2 = number of *N. gaeumannii* Lineage 2 isolates recovered from the site.

c CI = colonization index (average percentage of stomata occluded by pseudothecia).

d AFR (%) = average foliage retention (average percentage of needles remaining across four needle age classes).

e Total MLG not equal to sum of population totals due to shared MLGs. The genotypes of isolates collected in 2014 were analyzed previously in Bennett and Stone (2016).

* Estimated genotypic diversity from 1,000 bootstrap replicates with rarefaction (*n* ≥ 10).

### 3.2 Spatial distributions of *N. gaeumannii* lineages in relation to SNC severity

**Figure 2:**
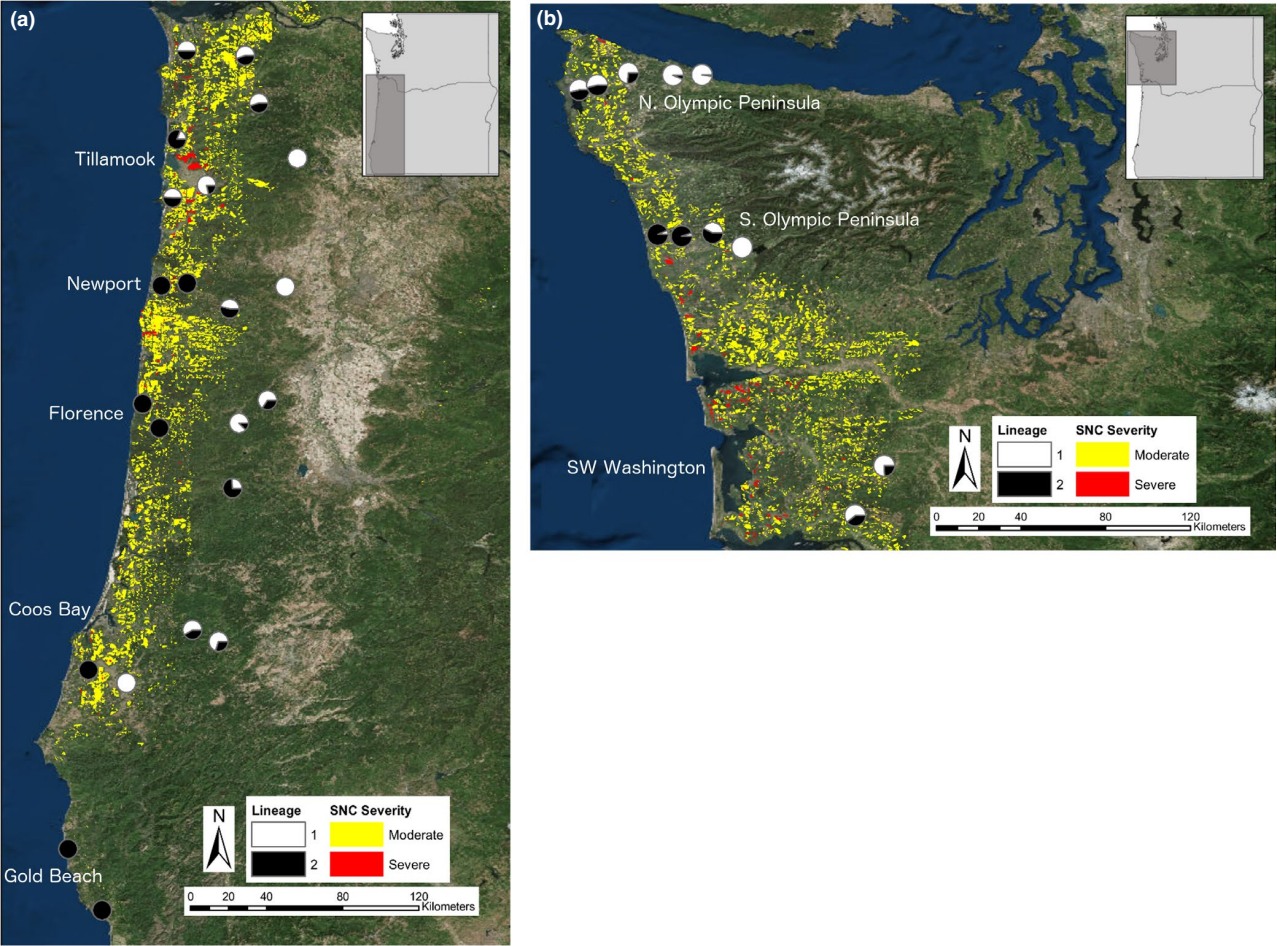
Swiss needle cast aerial survey maps with pie charts showing the geographic distributions of *Nothophaeocryptopus gaeumannii* lineages (a) 24 sites in western Oregon (for two of the pie charts, data from adjacent sites were pooled to avoid overlap), and (b) 11 sites in western Washington. Maps were produced with GIS data from the Washington Department of Natural Resources (Ramsey et al., 2015) and the Oregon Department of Forestry (https://www.oregon.gov/ODF/ForestBenefits/Pages/ForestHealth.aspx)

There was a strong association between the geographic distributions of *N. gaeumannii* Lineage 2 and SNC symptom severity assessed by aerial surveys (Figure 2). With few exceptions, sites nearest the coast had the highest proportions of Lineage 2 and occurred in areas where moderate‐to‐severe SNC symptoms were observed in the aerial surveys. Sites further inland, where Lineage 2 was generally rare or absent, had less severe SNC, or symptoms were not visible (Figure 2). However, aerial surveys did not detect symptoms of SNC in southwestern Oregon, where only Lineage 2 was isolated from foliage samples and Lineage 1 was not recovered (Figure 2).

### 3.3 Variation in geography, environment, and disease severity across sites

The 35 sampling sites in Oregon and Washington covered a latitudinal range from 42°N to 48°N, and a longitudinal range from –124.6°W to –123.3°W (Table 3). Sampling sites ranged from 16 to 595 m elevation and were located from 1.8 to 57.6 km inland (Table 3). Across sampling sites, mean average May–July dew point temperature (MADT) ranged from 7.3° to 10.5°, average spring/ early summer precipitation (PPT) varied from approximately 11.6–132.3 mm, mean summer temperature (TmSummer) ranged from 14.2° to 17.8°, and mean winter temperature (TmWinter) ranged from 4.1° to 10.3° (Table 3).

**Table 3:** Summary of variation in geography, disease severity, and environment across sites in Oregon and Washington from which Douglas‐fir foliage was sampled for isolation of *Nothophaeocryptopus gaeumannii*

Variable	Mean	*SD*	Min	Max
Disease				
CI^a^	19.2	9.4	3.7	42.8
AFR^b^	65.3	18.4	30.5	89.3
PL2^c^	0.5	0.4	0.0	1.0
Environment				
MADT^d^	9.2	0.8	7.3	10.5
PPT^e^	70.6	28.0	11.6	132.3
TmSummer^f^	15.9	0.9	14.2	17.8
TmWinter^g^	6.4	1.9	4.1	10.3
Geography				
Lat^h^	45.5	1.8	42.1	48.2
Long^i^	−123.9	0.4	−124.6	−123.3
Elev^j^	261.7	185.3	16.0	595.0
Dist^k^	26.0	18.3	1.8	57.6

a CI = colonization index (average percentage of stomata occluded by pseudothecia).

b AFR (%) = average foliage retention (average percentage of needles remaining across four needle age classes).

c PL2 = relative proportion of *Nothophaeocryptopus gaeumannii* Lineage 2 recovered from a site (number of Lineage 2 isolates/ total isolates).

d MADT = mean average dew point temperature (°C) (May–July year prior to sampling).

e PPT = average precipitation (mm) (May–July year prior to sampling).

f TmSummer = mean summer temperature (°C) (May–September year prior to sampling).

g TmWinter = mean winter temperature (°C) (November–March prior to sampling).

h Lat = latitude (decimal degrees).

i Long = longitude (decimal degrees).

j Elev = elevation (meters).

k Dist = distance inland (km).

Disease severity varied across the 34 sites for which disease severity data were available. Estimates of *AFR* ranged from 30.5% to 89.2%, with a mean of 65.3% (Table 2). *CI* ranged from 3.7 to 42.8, with a mean of 19.2. The most severe SNC symptoms occurred at sites in the vicinity of Tillamook, OR (Table 2), where the lowest *AFR* was at site T0‐3 (127 m elevation, 8.3 km inland), and the highest *CI* was at site T5‐3 (158 m elevation, 16.7 km inland; Table 2). The sites with the least severe SNC symptoms were at higher elevations and further inland. The highest AFR was at site N25‐5 (251 m elevation, 51 km inland), and the lowest *CI* was at T25‐2 (591 m elevation, 49.5 km inland; Table 2).

### 3.4 Correlations between environment, disease, and the genetic structure of *N. Gaeumannii* populations

**Figure 3:**
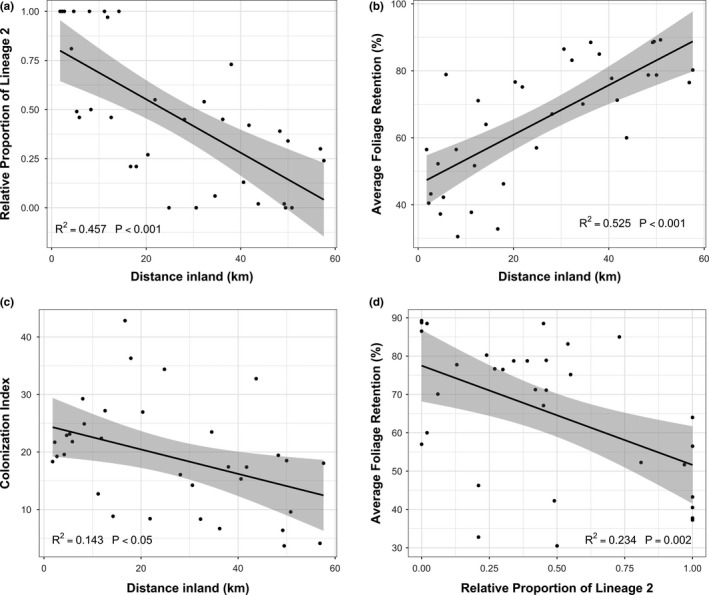
The relationships between distance inland (km) and (a) the relative proportion of isolates corresponding to *Nothophaeocryptopus gaeumannii* Lineage 2, (b) average foliage retention (AFR) (%), and (c) the colonization index (i.e., the average proportion of stomata occluded by *N. gaeumannii* pseudothecia). (d) The relationship between the relative proportion of Lineage 2 and average foliage retention when the distance inland (a major confounding variable) is not taken into account. Shaded regions represent 95% confidence intervals for the fitted lines

Pearson's coefficient (*r*) was calculated pairwise between the environmental, geographic, and disease variables in our dataset. *PL2* was negatively correlated with distance inland (Figure 3) and elevation (Table 4), and was positively correlated with mean winter temperature (Table 4). *AFR* was positively correlated with elevation and distance inland (Figure 3, Table 4). *CI* was negatively correlated with elevation and distance inland (Figure 3, Table 4) and was positively correlated with *MADT* (Table 4). *AFR* was negatively correlated with the relative proportion of *N. gaeumannii* Lineage 2 isolates recovered from the site (*PL2*) (Figure 3), *CI* (Figure 5), and *MADT* (Table 4).

**Table 4:** Pearson's correlation coefficients (*r*) for the relationships between SNC severity and each of the environmental and geographic variables used for this study

	CI^a^	AFR^b^	PL2^c^	MADT^d^	PPT^e^	TmSummer^f^	TmWinter^g^	Elev^h^	Dist^i^
CI	–								
AFR	−.59 (.000)	–							
PL2	−.03 (.857)	−.51 (.002)	–						
MADT	.65 (.000)	−.58 (.000)	.26 (.144)	–					
PPT	.32 (.062)	−.32 (.067)	.06 (.757)	.17 (.339)	–				
TmSummer	−.33 (.053)	.38 (.025)	−.21 (.236)	−.2 (.257)	−.28 (.109)	–			
TmWinter	.2 (.262)	−.48 (.004)	.51 (.002)	.6 (.000)	−.04 (.821)	.07 (.707)	–		
Elev	−.66 (.000)	.6 (.000)	−.38 (.027)	−.8 (.000)	−.19 (.272)	.39 (.023)	−.48 (.004)	–	
Dist	−.41 (.016)	.73 (.000)	−.69 (.000)	−.55 (.001)	−.14 (.423)	.49 (.004)	−.47 (.005)	.63 (.000)	–

Note

Numbers in parentheses represent computed *p*‐values for the correlation coefficient.

a Colonization index (average proportion of stomata occluded by pseudothecia).

b Average foliage retention (%).

c Relative proportion of Lineage 2 isolates from sample site (Lineage 2 isolates/total isolates).

d Mean average dew point temperature (°C).

e Spring/early summer precipitation (mm).

f Mean summer temperature (°C).

g Mean winter temperature (°C).

h Elevation (meters).

i Distance inland (km).

**Figure 4:**
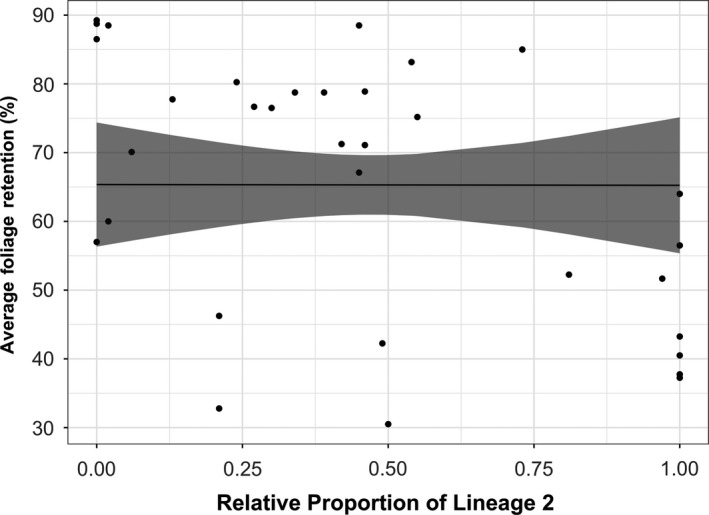
The relationship between average foliage retention (AFR) (%) and the relative proportion of *Nothophaeocryptopus gaeumannii* Lineage 2, after accounting for distance inland. Each point corresponds to one of the 34 sites for which disease severity data were available. The line corresponds to predicted values from the model when the distance inland is fixed at its mean (26 km), with the shaded region representing the 95% confidence intervals for the predicted values

**Figure 5:**
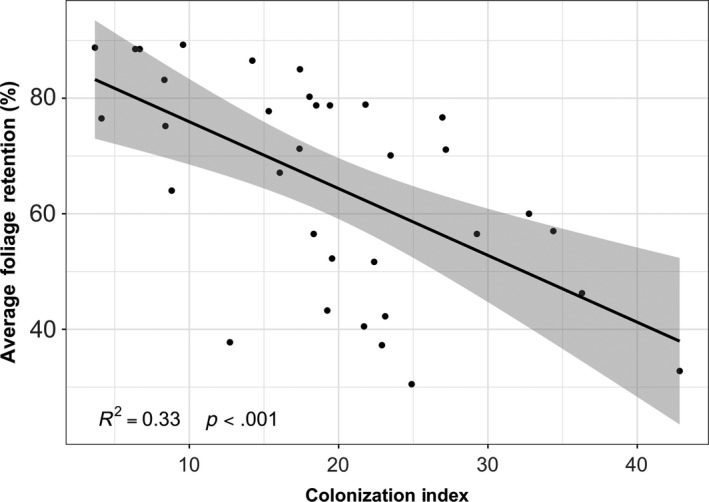
The relationship between average foliage retention (AFR) (%) and the colonization index (CI), an estimate of the average percentage of stomata occluded by pseudothecia of *Nothophaeocryptopus gaeumannii*. AFR and CI were measured for ten Douglas‐fir trees from each site. The data here reflect only the 34 sites for which disease severity data were available

The linear model described the site‐level *AFR* as a function of the relative proportion of *N. gaeumannii* Lineage 2 (*PL2*) recovered from the site, after accounting for distance inland (a variable that was strongly correlated with both *PL2* and SNC severity; Figure 4, Table 4). Although it initially appeared that there was a significant correlation between *AFR* and *PL2* (Figure 3d), this association was not significant when distance inland was held at its mean (25.95 km) (*t*_31_ = −0.01, *p* = .99) (Figure 4). There was a significant negative association between *AFR* and *CI*, but there also was considerable variation in *AFR* for a given value of *CI* (Figure 5), though foliage retention may be affected by a combination of environmental variables in addition to colonization by *N. gaeumannii*.

**Figure 6:**
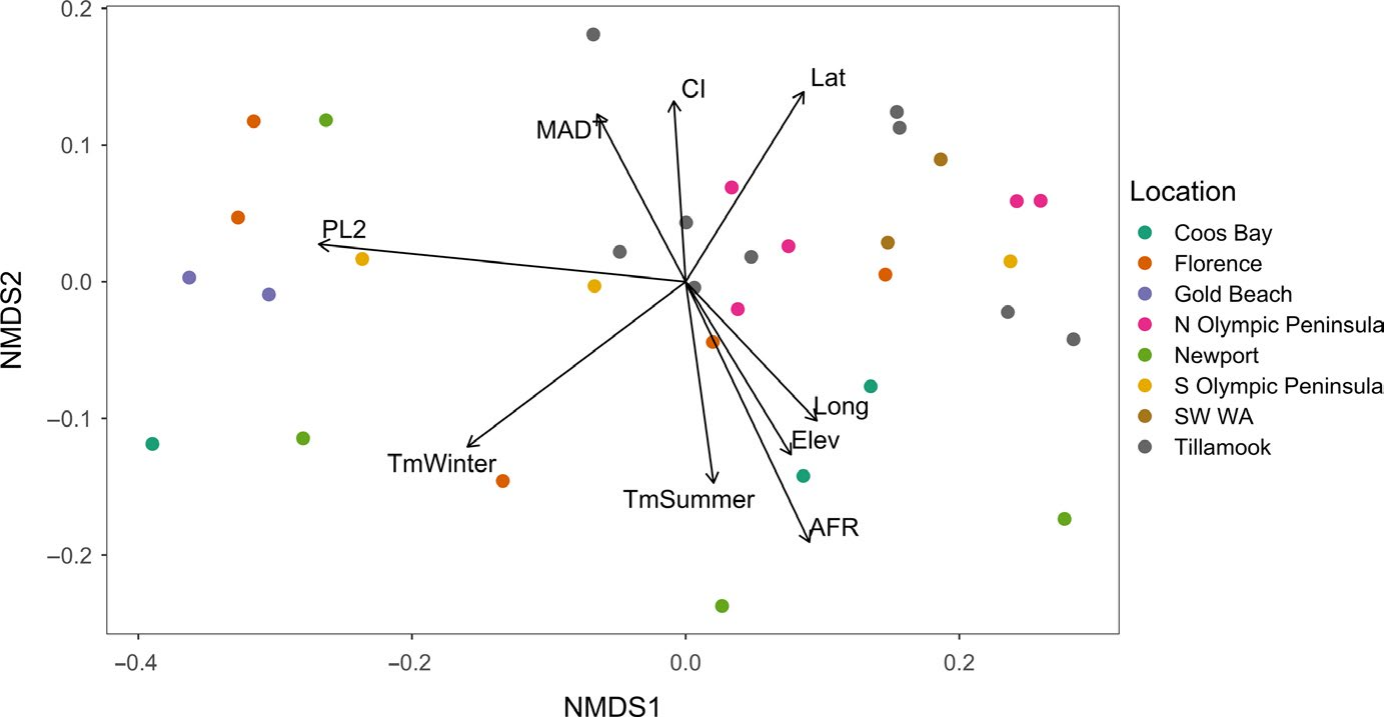
Nonmetric multidimensional scaling (NMDS) ordination based on the genetic distances between 33 sample sites calculated from multilocus SSR genotypes of *Nothophaeocryptopus gaeumannii* isolates. Joint plot vectors show correlations between environmental variables and the ordination. Only environmental variables with statistically significant correlations (*p* < .05) are shown. Vector labels correspond to the variables listed in Table 4. Final stress = 0.095. Locations in legend correspond to the sampling blocks. One Coos Bay site was removed from the ordination because it was an outlier, and one S Olympic Peninsula site was not included because disease severity estimates were not available

In initial runs of NMDS that included all of the 34 sites for which disease severity data were available, site CB5‐2 appeared as an outlier in the periphery of the plot and had a disproportionate influence on the ordination (see Section 4). For this reason, CB5‐2 was removed from subsequent analyses. The NMDS ordination of the 33 remaining sites for which disease severity data were available (Table 2) had a final stress of 0.095 (Figure 6). *PL2* had the strongest relationship with the NMDS ordination (*R* ^2^ = .947, *p* = .001) (Figure 6, Table 5). Therefore sites were aligned along NMDS axis 1 according to the relative proportion of the isolates recovered from the site that were identified as *N. gaeumannii* Lineage 2.

**Table 5:** Environmental, geographic, and disease variables used in the joint plot for the NMDS analysis based on the genetic distance *Nothophaeocryptopus gaeumannii*

Variable	Description	*R* ^2^ a	*p* b
CI	Colonization index (average percentage of stomata occluded by pseudothecia)	.228	.016
AFR	Average foliage retention (% foliage remaining across four needle age classes from branches in the midcanopy)	.569	.001
PL2	Relative proportion of Lineage 2 isolates in sample site (Number of Lineage 2 Isolates/Total Number of Isolates)	.947	.001
MADT	Mean average dew point temperature (°C) (May–July year prior to sampling)	.247	.015
PPT	Average precipitation (mm) (May–July year prior to sampling)	.147	.100
TmSummer	Mean summer temperature (°C) (May–September year prior to sampling)	.284	.012
TmWinter	Mean winter temperature (°C) (November–March prior to sampling)	.513	.001
Lat	Latitude (decimal degrees)	.342	.004
Long	Longitude (decimal degrees)	.253	.017
Elev	Elevation (meters)	.283	.009

a *R* ^2^ reflects the correlation between environmental variables and ordination scores.

b *p*‐values calculated from 999 permutations of the data.

In addition to the genetic differentiation across sites related to the divergence between lineages, there also appeared to be genetic variation between sites that was correlated with environment and SNC severity (Figure 6). Disease severity varied along NMDS axis 2 (Figure 6), and *AFR* was most strongly correlated with the ordination of genetic distance between the sites (*R* ^2^ = .569, *p* = .001) (Figure 6, Table 5). *CI* was inversely related to *AFR*, but was not correlated as strongly with the ordination (*R* ^2^ = .228, *p* = .016) (Figure 6, Table 5). Winter temperature (*TmWinter*) was the environmental variable most strongly correlated with the genetic differentiation between sites (*R* ^2^ = .513, *p* = .001), and with *PL2* (Figure 6, Table 5). Mean summer temperature (*TmSummer*) (*R* ^2^ = .284, *p* = .012) and average spring/early summer dew point temperature (*MADT*) (*R* ^2^ = .247, *p* = .015) had much weaker, but nonetheless significant, correlations with the ordination, while average precipitation in the spring and early summer was not significantly correlated with the ordination (Figure 6, Table 5). Latitude was the geographic variable with the strongest correlation with the ordination (*R* ^2^ = .342, *p* = .004), followed by elevation (*R* ^2^ = .283, *p* = .009), and longitude (*R* ^2^ = .253, *p* = .017) (Table 5). *AFR* and *TmSummer* were positively correlated with elevation and longitude, while *CI* was positively correlated with latitude and *MADT* (Figure 6, Table 5).

## 4 DISCUSSION

The population of *N. gaeumannii* in Oregon and Washington is diverse with a genetic structure that reflects the presence of two strongly differentiated noninterbreeding lineages (Figure 1) (Bennett & Stone, 2016; Winton et al., 2006). These characteristics are consistent with the presumed endemism of *N. gaeumannii* in northwestern North America (Hansen et al., 2000; Winton et al., 2006). The presence of repeated multilocus genotypes (i.e., clones) within the two lineages (Tables 1 and 2) likely reflects reproduction via homothallism, as *N. gaeumannii* is not known to reproduce asexually (Winton et al., 2006; Winton, 2001). This reproductive mode likely contributes to the genetic structure of *N. gaeumannii* populations, but the level of genetic variation observed suggests that outcrossing also occurs.

Although the two *N. gaeumannii* lineages have overlapping distributions, it appears that each is adapted to slightly different environmental conditions and thus exhibit different habitat distributions that are determined, directly or indirectly, by climate. The spatial distribution of *N. gaeumannii* Lineage 2 corresponded to the regions where SNC symptoms were most severe suggesting that some variation in aggressiveness between the two lineages might exist, as suggested by previous authors (Winton et al., 2006). However, we found no evidence to suggest that *N. gaeumannii* Lineage 2 is more aggressive than Lineage 1. After accounting for distance inland, a confounding variable in the relationship between *PL2* and *AFR*, there was no significant correlation between Lineage 2 and SNC severity. Thus, the association between Lineage 2 and greater defoliation can be attributed to overall abundance of *N. gaeumannii* rather than dominance of Lineage 2 genotypes.

The separation of sites along axis 2 in the NMDS analysis (Figure 6) reflects genetic differentiation between the isolates collected at sites with severe disease (higher CI and lower AFR) and those collected at sites with lower disease severity (lower CI and higher AFR). This genetic differentiation was not related to the relative abundances of Lineages 1 and 2. This suggests that some variation in aggressiveness among isolates exists that is not related to the genetic differentiation between the two lineages. These observations also suggest that adaptation to local climate or natural selection for advantageous genotypes has occurred in the geographic regions where SNC is most severe. Whether *N. gaeumannii* populations in the coastal SNC epidemic zone are in fact more aggressive or have increased fitness (and thus cause more severe symptoms) is still unclear and should be the focus of future studies.

The relative abundances of the lineages varied along a west–east gradient, with Lineage 2 more abundant relative to Lineage 1 in sites near the coast and decreasing in relative abundance further inland. At sites approximately 40–56 km inland, Lineage 2 was supplanted entirely by Lineage 1 in some sites. The correspondence between the geographic distributions of *N. gaeumannii* Lineage 2 and severe SNC symptoms in western Oregon and Washington (Figure 2) initially suggested a causal relationship between the relative abundance of this lineage and disease severity. At the landscape level, the SNC symptoms documented by aerial surveys were most prevalent along the western slopes of the Coast Ranges, where Lineage 2 was generally more abundant, or where the two lineages coexisted within ~30 km of the coast (Figure 2). This trend was observed in the low‐elevation forests along the western slopes of the Coast Ranges in Oregon and Washington from Coos Bay, OR to the northern Olympic Peninsula (Figure 2, Table 2). Further inland, Lineage 2 was generally less abundant, or absent, and SNC symptoms were generally less severe or not detected (Figure 2).

The observation that the regions where distributions of the two *N. gaeumannii* lineages overlap correspond to the regions with severe SNC is in agreement with previous studies (Winton et al., 2006). The apparent association between *PL2* and *AFR* when distance inland was not included in the model (Figure 3, Table 4) also supports the interpretation by Winton et al. (2006) that SNC symptoms were more severe in Douglas‐fir stands with higher proportions of Lineage 2. However, *PL2* was not correlated with the colonization index (*CI*) (Table 4), the variable that should have the strongest mechanistic relationship with foliage retention (Manter et al., 2005, 2003). Given the current understanding of the mechanisms of disease in this pathosystem, where premature foliage loss associated with SNC results from cumulative occlusion of the stomata by the pseudothecia of *N. gaeumannii* (Manter et al., 2000, 2005, 2003; Stone, Capitano, et al., 2008), the most plausible mechanism by which Lineage 2 could cause more severe defoliation than Lineage 1 would be by colonizing the host needle more rapidly and subsequently producing more abundant pseudothecia relative to Lineage 1. Our analyses do not support such a mechanism.

Lineage 1 was not isolated from the Douglas‐fir foliage samples collected at the two southernmost sites in Oregon near Gold Beach (Figure 2). These sites were composed exclusively of *N. gaeumannii* Lineage 2. To date, aerial surveys have documented very low incidence of SNC symptoms along the Oregon coast south of Port Orford (Figure 2), although Douglas‐fir is abundant (Lavender & Hermann, 2014) and *N. gaeumannii* is relatively common. The fact that we only recovered isolates of *N. gaeumannii* Lineage 2 from those sites, yet symptoms of SNC are not observed there, provides further evidence that Lineage 2 is not in fact associated with increased SNC severity.

The NMDS ordination of sites in relation to genetic distance revealed strong spatial genetic differentiation between inland and coastal sample sites (Figure 6). This approach allowed for a visualization of relationships between genetic variation and environmental, geographic, and disease variables. The spatial distribution of genetic variation was strongly correlated with *Tmwinter*, *Tmsummer*, and *MADT* in the year prior to sampling, but not *PPT* (Figure 6, Table 5). Precipitation in the western Coast Ranges is generally abundant during the period of sporulation and infection by *N. gauemannii*, so spatial variation in spring/summer precipitation has not been a useful variable for modeling *N. gaeumannii* abundance in this region (Manter et al., 2005). The distribution of *N. gaeumannii* Lineage 2 was most strongly correlated with *Tmwinter* (Figure 6, Table 5). Foliage retention varied most strongly with *Tmsummer*, elevation, and longitude, and was negatively correlated with *MADT* (Figure 6, Table 5). Sites with lower *AFR* values and higher *CI* values generally occurred at lower elevations nearest the coast where summers were cooler and spring/early summer dew point temperatures were warmer (Figure 6). The *N. gaeumannii* populations occurring in these sites appeared to be genetically differentiated from the higher elevation inland sites where the winters were colder, summers were hotter, and *AFR* was greater (Figure 6).

The environmental variable most strongly correlated with the spatial distribution of Lineage 2 (*Tmwinter*) is also strongly correlated with *MADT*, the variable most strongly associated with needle colonization by both lineages (Table 4). This suggests that the environment most conducive to needle colonization is also optimal for *N. gaeumannii* Lineage 2, but that Lineage 1 has a broader range of environmental tolerance than Lineage 2. These apparent differences in environmental tolerance may be related to the underlying causes of reproductive isolation and evolutionary divergence between the two lineages. These results also suggest that the distribution of genetic variability within and between *N. gaeumannii* populations is influenced by environmental factors, possibly due to the influences of natural selection and local adaptation. Although the SSR markers used here are presumed to be selectively neutral, and thus are not directly influenced by the environment, they provided a tool with which to detect genetic differentiation between populations that may be associated with adaptation to local climate.

Previous studies have identified significant associations between environmental variables, colonization by *N. gaeumannii*, and SNC symptoms (Coop & Stone, 2010; Hansen et al., 2000; Lee et al., 2013, 2017; Manter et al., 2005; Rosso & Hansen, 2003; Shaw, Woolley, & Kanaskie, 2014; Stone, Coop, et al., 2008; Stone et al., 2007; Watt et al., 2010; Zhao et al., 2012, 2011). Thus it was expected that *Tmwinter* and variables associated with moisture availability would be correlated with the abundance of *N. gaeumannii*, measured here as *CI*. However, in the present study *Tmwinter* was significantly associated with *AFR* (*r* = −.48, *p* < .05) but not *CI* (*p* > .05), and *PPT* was not significantly associated with either *AFR* (*p* > .05) or CI (*p* > .05) (Table 4). However, spring and early summer dew point temperature (*MADT*) was significantly correlated with *CI* suggesting that *N. gaeumannii* colonization is highest where moisture is abundant at warmer temperatures (Table 4).

Given that the occlusion of stomata causes the foliage loss associated with SNC, we expected the correlation between *AFR* and *CI* to be stronger, as reported in previous studies (Manter et al., 2003; Watt et al., 2010). In our dataset, there was considerable variation in *AFR* for a given value of *CI*, suggesting genetic variation in host tolerance (i.e., some host genotypes can tolerate higher levels of infection before needle abscission occurs) and/or that *AFR* may be affected by environmental factors in combination with SNC severity. This variation in *AFR* in relation to *N. gaeumannii* abundance has been investigated for SNC in coastal Douglas‐fir (Temel, Johnson, & Stone, 2004). In that study, the authors concluded that some genetic variation in host tolerance reflected the historical SNC pressure in the environment where the host genotype evolved (Temel et al., 2004). Generally, Douglas‐fir provenances from regions where rainfall and humidity are low are less tolerant of SNC because natural selection has not favored tolerant individuals where disease pressure is historically low (Hood, 1982; Mcdermott & Robinson, 1989; Temel et al., 2004). The variation in *AFR* observed in our study may also reflect some direct interaction between environment and disease; SNC is one of several factors that may affect *AFR*. It seems reasonable to assume that some unfavorable environmental conditions may lead to greater foliage loss at lower levels of infection and that favorable conditions may allow hosts to maintain a healthy level of foliage with higher levels of infection.

Distance inland and elevation are two geographic variables that had significant associations with both *PL2* and disease severity in our data. While these geographic variables may not directly affect biological processes that influence *N. gaeumannii* abundance or SNC severity, their effects on the biological system are a reflection of interacting climatic and environmental factors along a gradient from the low‐elevation forests along the western slopes of the Coast Range to the higher elevation forests further inland along the Coast Range (Hansen et al., 2000; Shaw et al., 2014; Zhao et al., 2011). In fact, distance inland and elevation were collinear with several environmental variables examined in this study. We therefore used distance inland as a proxy for continentality, a complex combination of multicollinear environmental factors that vary along this geographic gradient (Zhao et al., 2011). A synthesis of the results from this study provides a framework for understanding the factors influencing SNC severity and the genetic structure of *N. gaeumannii* populations. In general, the sites with the most severe SNC and the highest *PL2* were at low elevations (<300 m) within ~20 km of the coast along the western slopes of the Oregon and Washington Coast Ranges where winter temperatures were the warmest (~6–10°C), summers were coolest (~14–16°C), and the dew point temperatures (the temperature at which the air was saturated with water vapor) were warmest (~9–11°C). These observations are in agreement with previous studies that demonstrated that warmer winter temperatures and abundant moisture in the spring/early summer, though not necessarily as precipitation, favor *N. gaeumannii* abundance and thus SNC severity (Lee et al., 2013, 2017, 2016; Manter et al., 2005; Stone, Coop, et al., 2008; Stone et al., 2007; Watt et al., 2010). Our observations are also in agreement with studies that suggested that high summer temperatures inhibit the growth of *N. gaeumannii* (Rosso & Hansen, 2003), resulting in lower SNC severity (Lee et al., 2017; Manter et al., 2005; Stone, Coop, et al., 2008; Stone et al., 2007; Watt, Stone, Hood, & Manning, 2011; Watt et al., 2010; Zhao et al., 2012). Climate change (i.e., increasingly warmer winter temperatures) will likely exacerbate SNC severity in the western Coast Range leading to an intensification of symptoms in areas already affected by SNC, as well as an expansion of the area affected by SNC (Coop & Stone, 2010; Lee et al., 2017; Stone, Coop, et al., 2008; Watt et al., 2011, 2010).

One site near Coos Bay, Oregon (CB5‐2) did not fit with the overall trends observed for the geographic distributions of the two lineages in relation to coastal proximity. This site was within 25 km of the coast, but all of the isolates recovered from two separate trees were Lineage 1 (Table 2, Figure 2). This was unexpected, considering that the isolates sampled from a site just a few kilometers to the west were all Lineage 2, and both lineages were recovered from the closest sites to the northeast (Figure 2, Table 2). This suggests that little gene flow is occurring between these sites, even though they are only a few kilometers apart. There were only eight isolates sampled from this site, and thus the estimated *PL2* (and the estimated relationships between *PL2* and other variables) may have been affected by sampling bias. The sample of eight isolates from this site had MLGs that were very dissimilar from those collected from nearby sites. Because all isolates collected from CB5‐2 were Lineage 1, but Lineage 2 should have been relatively abundant given the site location, this site disproportionately influenced the fitting of the geographic variables in the NMDS, especially latitude. This suggests that the isolates at CB5‐2 were more similar to sites further north, even though this site is near the southern coast of Oregon.

The results of our analyses suggest that climate influences the population structure of this ecologically important Douglas‐fir pathogen. Not only does the environment influence SNC severity through direct effects on growth rate and reproduction of *N. gaeumannii*, but environmental variables were also correlated with spatial genetic differentiation in the *N. gaeumannii* population. This suggests that climate may play a role in the evolutionary divergence of these cryptic fungal lineages. Climate change has the potential to be a driver of further genetic change in *N. gaeumannii* populations.

The strong genetic differentiation between the two *N. geaumannii* lineages observed in this study is in agreement with the results of previous analyses that suggested that the lineages were reproductively isolated (Bennett & Stone, 2016; Winton et al., 2006). Although the reproductive incompatibility of these two lineages cannot be definitively demonstrated, as this fungus does not produce ascomata in culture, the divergence observed suggests that the two lineages constitute separate ecological species. However, designation of Lineage 1 and Lineage 2 as distinct species will require phylogenetic or phylogenomic analyses to evaluate the degree of evolutionary divergence between the lineages.

The scope of this study was limited due to the use of highly variable neutral markers, which are useful for identifying populations and estimating genetic differentiation but cannot be used for analyses of evolutionary processes such as natural selection and adaptation. Thus, a thorough demonstration of the role of environment in structuring populations of *N. gaeumannii* was not possible with the available molecular tools. Recent advances in population genomics have enabled the identification of the molecular mechanisms involved in adaptation to local environmental conditions. This analytical framework could be applied to *N. gaeumannii* as a next step toward elucidating the environmental factors contributing to spatial genetic variability in its populations and identifying the mechanistic influences of these environmental factors on its biology.

## CONFLICT OF INTEREST

The authors have no competing interests to declare.

## AUTHOR CONTRIBUTIONS

PIB and JKS designed the study, PIB performed all laboratory work and data analyses, produced all figures, and drafted the manuscript. Editing of the manuscript was performed collaboratively by PIB and JKS.

## Data Availability

Microsatellite genotypes, environmental data, and R scripts for reproducing analyses and figures: Dryad https://doi.org/10.5061/dryad.2p7r5g6.
